# Supplementary material for: Constitutive STAT5 activation regulates Paneth and Paneth-like cells to control Clostridium difficile colitis
Source: Life Sci Alliance. 2019 Apr 4;2(2):e201900296. doi: 10.26508/lsa.201900296 (PMC6451325; doi:10.26508/lsa.201900296)
Supplement: Supplementary file 1 [file LSA-2019-00296_TableS1.docx]

**Table S1.** Antibodies for FACS Analysis.

| Antibodies | Manufacturer | Surface, intracellular or nuclear staining |
| --- | --- | --- |
| PE-Cy7-anti-pYSTAT5(pY6494) | BD Pharmingen | Intracellular |
| APC-anti-pYSTAT5(pY694) | BD Pharmingen | Intracellular |
| Percp-anti-pYSTAT5(pY694) | BD Pharmingen | Intracellular |
| PE-anti-HumanCD2 | BD Pharmingen | Surface |
| PE-anti-CD24 | BD Pharmingen | Surface |
| APC-anti-CD44 | BD Pharmingen | Surface |
| PE-anti-Ki67 | eBioscience | Nuclear |
| APC-anti-BrdU | BD Pharmingen | Nuclear |
