# Supplementary material for: Constitutive STAT5 activation regulates Paneth and Paneth-like cells to control Clostridium difficile colitis
Source: Life Sci Alliance. 2019 Apr 4;2(2):e201900296. doi: 10.26508/lsa.201900296 (PMC6451325; doi:10.26508/lsa.201900296)
Supplement: Supplementary file 2 [file LSA-2019-00296_TableS2.docx]

**Table S2.** PCR Primers for Genotyping Mutant Mice.

| Primers for Genotyping PCR | Sequences |
| --- | --- |
| *Stat5f/f* | 5'-GAA AGC ATG AAA GGG TTG GAG-3’ |
|  | 5’-AGC AGC AAC CAG AGG ACT AC-3' |
|  | 5'-AAG TTA TCT CGA GTT AGT CAG G-3' |
| *icS5* | 5'-AGG CGA CCA TCA TCA GCG AGC-3' |
|  | 5'-GAA TGG AGA AAT CTC GCG TCG-3' |
| *VilCre*ER | 5’-CAA GCC TGG CTC GAC GGC C-3’ |
|  | 5’-CGC GAA CAT CTT CAG GTT CT-3’ |
| *VilCre* | 5’-GTG TGG GAC AGA CAA ACC-3’ |
|  | 5’-ACA TCT TCA GGT TCT GCG GG-3’ |
| *Lgr5Cre*ER | 5’-CAC TGC ATT CTAGTT GTGG-3’ |
|  | 5'-CGG TGC CCG CAG CGAG-3’ |
| *Rs26Cre*ER;mT/mG | 5'-TCA ATG GGC GGG GGT CGT T-3' |
|  | 5'-CTC TGC TGC CTC CTG GCT TCT-3' |
|  | 5'-ATG TAC ATT TTG TAC TCA CAG AGA TGG A-3' |
| *Rs26Cre*ER*;*tdtomato | 5'-AAG GGA GCT GCA GTG GAG TA-3' |
|  | 5'-CCG AAA ATC TGT GGG AAG TC-3' |
|  | 5'-GGC ATT AAA GCA GCG TAT CC-3' |
|  | 5'-CTG TTC CTG TAC GGC ATG G-3' |
| *Rs26Cre*ER*;*LacZ | 5`-TTC CAT GTT GCC ACT CGR TTT A-3` |
|  | 5`-GTT TCG GGT TTT CGA CGT TCA G-3` |
