# Supplementary material for: Constitutive STAT5 activation regulates Paneth and Paneth-like cells to control Clostridium difficile colitis
Source: Life Sci Alliance. 2019 Apr 4;2(2):e201900296. doi: 10.26508/lsa.201900296 (PMC6451325; doi:10.26508/lsa.201900296)
Supplement: Supplementary file 3 [file LSA-2019-00296_TableS3.docx]

**Table S3.** Primers for Quantitative Real-time PCR.

|  | Forward | Reverse |
| --- | --- | --- |
| *Defensinb1* | AGGTGTTGGCATTCTCACAAG | GCTTATCTGGTTTACAGGTTCCC |
| *Defensinb3* | GCATTGGCAACACTCGTCAGA | CGGGATCTTGGTCTTCTCTA |
| *Defensinb10* | TTGTCCTGGTAATAGCAGGTTTATGA | CGGAGATTCTCTGGGTGACAGT |
| *Muc2* | CCTTAGCCAAGGCTCGGAA | GGCCCGAGAGTAGACCTTGG |
| *Reg3γ* | TTCCTGTCCTCCATGATCAAA | CATCCACCTCTGTTGGGTTC |
| *Sox9* | TGCCCATGCCCGTGCGCGTCAA | CGCTCCGCCTCCTCCACGAAGGGTCT |
| *Lysozyme* | TGACATCACTGCAGCCATAC | TGGGACAGATCTCGGTTTTG |
| *GAPDH* | GGTGGGTGGTCCAAGGTTTC | TGGTTTGACAATGAATACGGCTAC |
